# Hsa_circ_0002348 regulates trophoblast proliferation and apoptosis through miR-126-3p/BAK1 axis in preeclampsia

**DOI:** 10.1186/s12967-023-04240-1

**Published:** 2023-07-28

**Authors:** Jizi Zhou, Ying Zhao, Ping An, Huanqiang Zhao, Xiaotian Li, Yu Xiong

**Affiliations:** 1 Obstetrics and Gynecology Hospital, Fudan University, Shanghai, China; 2 Shanghai Key Laboratory of Female Reproductive Endocrine Related Diseases, Shanghai, China; 3 Institute of Biomedical Sciences, Fudan University, Shanghai, China

**Keywords:** CircRNA, Preeclampsia, Trophoblast, Proliferation, Apoptosis, miR-126-3p/BAK1 axis

## Abstract

**Background:**

Preeclampsia is a common pregnancy complication characterized by high blood pressure and damage to organs. Abnormal placenta and vascular function can lead to preeclampsia. Accumulating evidence has suggested a potential link between circular RNAs (circRNAs) and preeclampsia. As a placenta and endothelial-expressed circRNA, hsa_circ_0002348, may be promising to be the novel molecular target for preeclampsia. However, the function and mechanism of hsa_circ_0002348 in preeclampsia has not been elucidated.

**Materials and methods:**

An overlap analysis of two circRNA profiles from placenta and endothelial cells was used to identify a functionally unknown circRNA, hsa_circ_0002348. Quantitative real-time PCR (qRT-PCR) and in situ hybridization (ISH) were used to detect its expression in the trophoblast cells and placental tissues. The mouse model of lipopolysaccharide (LPS)-induced preeclampsia was established to determine the in vivo role of hsa_circ_0002348. RNA immunoprecipitation (RIP), Luciferase reporter assay, qRT-PCR, western blot, gain- and loss-of-function and rescue experiments were conducted to uncover the role of hsa_circ_0002348 and its interaction with miR-126-3p and BAK1 in regulating trophoblast proliferation and apoptosis. Fluorescence in situ hybridization (FISH) and Immunohistochemistry (IHC) were performed to examine the expression of miR-126-3p and BAK1 in mice and human placentas, respectively.

**Results:**

Hsa_circ_0002348 was significantly increased in the preeclampsia placentas, and positively correlated with the severity of preeclampsia patients’ clinical manifestations. Its overexpression exacerbated preeclampsia-like features in the mouse model of LPS-induced preeclampsia. Functionally, hsa_circ_0002348 was found to inhibit trophoblast proliferation and promote trophoblast apoptosis. Mechanistically, hsa_circ_0002348, as an endogenous miR-126-3p sponge, upregulated the expression of BAK1. Additionally, both hsa_circ_0002348 knockdown and miR-126-3p overexpression enhanced the mammalian target of rapamycin (mTOR) and ERK1/2 signaling pathway.

**Conclusions:**

Hsa_circ_0002348 might be a novel regulator of trophoblast proliferation and apoptosis through miR-126-3p/BAK1 axis in preeclampsia, which may serve as a potential target for detecting and treating preeclampsia.

**Supplementary Information:**

The online version contains supplementary material available at 10.1186/s12967-023-04240-1.

## Introduction

Preeclampsia is one of the most common obstetric complications defined as hypertension and proteinuria developing after 20 weeks of gestation, affecting almost 2.5–3% of pregnancies [1]. Once preeclampsia causes severe maternal multisystemic damage, termination of pregnancy is the only effective treatment [2]. More than that, a long-term threat to health persists in mother and offspring [2, 3]. The exact cause of preeclampsia is unknown, but it has been widely accepted that preeclampsia originates from abnormal placentation [4]. During normal placental formation, different types of trophoblast cells work together to proliferate, differentiate, invade and replace endothelial cells, ultimately completing the remodeling of the spiral arteries and establishing an adequate maternal–fetal circulation [5]. As another biologic function, trophoblast apoptosis is also involved in this process, contributing to normal placenta development [6]. Overall, when trophoblast cells do not function properly, it can lead to insufficient blood flow between the mother and fetus, which could contribute to the occurrence of preeclampsia. Therefore, understanding the molecular mechanism underlying trophoblast functions is an important area of research, as it may help develop new strategies for detection and treatment of this serious pregnancy complication.

Circular RNAs (circRNAs), a newly found series of non-coding RNAs (ncRNAs) characterized by their loop structure without 5′–3′ polarities and polyadenylated tails, are expressed in both the nucleus and cytoplasm of eukaryotic cells [7]. A growing body of studies has reported that circRNAs involve in various pathobiological process, serving as disease biomarkers, competing endogenous RNA (ceRNA) [8], interacting with RNA binding proteins (RBPs) [9], and being translated into peptide [10]. In terms of preeclampsia, some maternal circulating blood derived circRNAs [11–13] are initially identified to be used as biomarkers for the prediction of preeclampsia. Additionally, some investigators have confirmed hundreds of circRNAs that are altered in the placentas of women with preeclampsia compared to healthy controls and established associated ceRNA networks [14]. Thus, further research on the mechanism of circRNAs in preeclampsia is feasible and necessary. In order to explore the novel circRNA target of preeclampsia, we performed an intersection analysis of two circRNAs expression profiles derived from placenta and endothelial cells, respectively, and determined a placenta and endothelial-expressed circRNA, hsa_circ_0002348. Nevertheless, there is no study previously reported regarding its role and regulation mechanism in the pathogenesis of preeclampsia.

Here, we reported that hsa_circ_0002348 was significantly upregulated in the placental tissues of preeclampsia patients and exacerbated preeclampsia-like features in the mouse model of LPS-induced preeclampsia. Mechanistically, hsa_circ_0002348 inhibited trophoblast proliferation and promoted apoptosis through competitively binding to miR-126-3p, thus regulating the expression of BAK1. Conclusively, our findings identified a potential circRNA functioning in preeclampsia, which may help to develop more effective approaches for its detection and treatment.

## Materials and methods

### Patients, samples collection

The patients were diagnosed of preeclampsia according to the American College of Obstetricians and Gynecologists (ACOG) as follows: systolic blood pressure (SBP) of ≥ 140 mmHg and/or diastolic blood pressure (DBP) of ≥ 90 mmHg with proteinuria of ≥ 300 mg/day (or a protein/creatinine ratio of ≥ 0.3 mg/dl or proteinuria of ≥ 1+) after 20 weeks of gestation. Severe preeclampsia was defined by one or more of the following criteria: maternal blood pressure ≥ 160/110 mmHg; severe clinical symptoms such as visual disturbances, pulmonary edema, epigastric or right upper quadrant pain, fetal growth restriction, oligohydramnios. The pregnant women were asked to take a rest of at least five-minutes with the relaxed limbs and an appropriate cuff size, before measuring blood pressure. We usually measured blood pressure in the right upper limb, requiring the cuff to be at the same level as the heart. Urine samples were required to be retained for clean catch midstream.

The placental tissues were obtained from 42 healthy pregnant women, 22 pregnant women with preeclampsia and 27 pregnant women with severe preeclampsia after delivery, a list made of their general characteristics (Additional file 1: Table S1). Among the three groups, no significant difference was observed in maternal age, body mass index (p > 0.05). The group of severe preeclampsia showed the earlier gestation of delivery and the lighter birth weight when compared with the control group (p < 0.001) and the group of mild preeclampsia (p < 0.05). Approved by the Ethics Committee of the Obstetrics and Gynecology Hospital of Fudan University (2021-198), the present study was permitted to collect all the samples from the participants with a written informed consent.

### RNA extraction and quantitative reverse transcription polymerase reaction (qRT-PCR)

Total RNAs of the tissues and cells were extracted using TRIzol regent (Invitrogen, USA) to be purified following the manufacturer’s instructions. The circRNA and mRNA were reversely transcribed into cDNA using PrimeScript II 1stStrand cDNA Synthesis Kit (Takara, Japan), while the miRNAs were reversely transcribed into cDNA with TaqMan microRNA Reverse Transcription Kit (ThermoFisher, USA). Based on Roche480Real-Time PCR system, quantitative real-time PCR was performed using a SYBR®Premix Ex Taq II (Takara, Japan). β-actin served as internal control for circRNAs and mRNAs. The relative expression of RNAs was calculated via 2^−∆∆Ct^ method with a log2-transformed step to convert the fold-change values into a format that fitted the downstream analysis. A list was made of the primers sequences in qRT-PCR (Additional file 2: Table S2), all of which were synthesized by Genepharma Co., Ltd. (Shanghai, China).

### In situ hybridization for circRNA detection

In situ hybridization (ISH) was conducted with BaseScope™ Reagent Kit, V2-RED (#323900-USM, Advanced Cell Diagnostics (ACD) Hayward, CA). Hsa_circ_0002348 junction site-targeting/-non targeting labeled probes conjugated to horseradish peroxidase (HRP) from ACD (#701041 & #701021). According to the manufacturer’s protocol, 5 μm of formalin-fixed paraffin-embedded (FFPE) human placenta sections were baked to be deparaffinized, before pretreated with hydrogen peroxide, followed by target retrieval performed and hydrophobic barrier created using the Immedge™ hydrophobic barrier pen. The dried slides were placed on the slide rack to be incubated with Protease Plus at 40 °C for 30 min in the HybEZ^TM^system (ACD). After that, the slides were hybridized with probes (hsa_circ_0002348) at 40 °C for 2hs in HybEZ^TM^system, followed by signal amplification performed as follows: AMP 1 for 30 min at 40 °C; AMP 2 for 15 min at 40 °C; AMP 3 for 30 min at 40 °C; AMP 4 for 15 min at 40 °C; AMP 5 for 30 min at RT; and AMP 6 for 15 min at RT. Following counterstain with hematoxylin, chromogenic detection was performed using BaseScope™ Fast RED. According to RNAscope standards (score of 4+), BASEScope intensity was quantified by the numbers of positive cells that exhibited at least 10 red puncta.

### Cell culture and transfection

The human first-trimester extravillous trophoblast (EVT)-derived cell line, HTR8/SVneo [15], which was purchased from American Type Culture Collection (ATCC, Manassas, VA, USA), was cultured in the RPMI 1640 media (Life Technologies, Shanghai, China) with 10% fetal bovine serum (FBS) (Life Technologies, Shanghai, China), with 100 μg/ml streptomycin and 100 U/ml penicillin as supplements. These cells were maintained under standard culture conditions of 5% CO_2_ and 37 °C.

Small interfering RNAs (siRNAs) specific for the back-splice junction sequences of hsa_circ_0002348, miR-126-3p mimics, and their corresponding negative control oligonucleotides, were constructed by Genepharma Co. Ltd. (Shanghai, China), with a list made of the sequences of siRNA (Additional file 3: Table S3). Following the manufacturer’s instructions, the transfection of these oligonucleotides was performed using Lipofectamine 2000 (Invitrogen, USA).

### Actinomycin D, RNase R treatment and nuclear-cytoplasmic fractionation

To determine the expression levels of hsa_circ_0002348 and its host gene, cysteine rich transmembrane BMP regulator 1 (CRIM1) mRNA, respectively, HTR8/SVneo cells were treated with Actinomycin D and RNase R to effectively digest linear RNA. For Actinomycin D treatment, 2 mg/ml of actinomycin D or dimethylsulphoxide (Sigma-Aldrich, St. Louis, MO, USA) was added, as a negative control, to the cell culture medium. For ribonuclease R (RNase R) treatment, total RNA (4 μg) was mixed with 20 units of RNase R (Epicenter; USA, RNR07250) in the presence of 1× Reaction Buffer for 30 min at 37 °C. The nuclear and cytoplasmic fractions were isolated with the Nuclear and Cytoplasmic Extraction Kit (Cwbio, China) following the manufacture’s protocol. GAPDH, U1, MALAT1 and NEAT1 were employed as the positive controls for cytoplasmic and nuclear fractions, respectively.

### CCK8 assay

Cell Counting Kit-8 (CCK8) assay (RiboBio, Shanghai, China) was performed to assess cell proliferation. The cells at 1 × 10^3^ were seeded into 96-well plates to be treated with 10ul of CCK-8 solution at the time intervals of 0, 24, 48 and 72 h after 2 h-incubation at 37 °C. The absorbance at 450 nm was read with an enzyme immunoassay analyzer (Thermo Fisher Scientific, Inc., Waltham, MA, USA). This experiment was repeated thrice.

### Colony formation assay

Following 24 h-transfection, HTR8/SVneo cells were seeded into 6-well plates, with 500 per well. Incubated for 2 weeks, the cells were fixed in methanol and stained with 0.1% crystal violet.

### Cell apoptosis assays

With Annexin V-FITC/Propidium Iodide Apoptosis Detection Kit (BD Pharmingen, New York, USA, #556547), cell apoptosis was assessed according to the manufacturer’s instructions. Transfected for 48 h, the cells were collected to be stained with Annexin V-FITC and propidium iodide (PI), before analyzed by fluorescence-activated cell sorting using FACS Canto II (BD Biosciences, San Jose, CA, USA). The apoptosis data were calculated based on FlowJo V10 software (Tree Star, San Francisco, CA, USA). Those of quadrant 4 with FITC Annexin V and PI negative were considered to be viable; those of quadrant 3 with FITC Annexin V positive and PI negative, to be in early apoptosis; those of quadrant 2 with FITC Annexin V and PI positive, to be in late apoptosis; and those of quadrant 1 with FITC Annexin V negative and PI positive, to be necrotic. The rates of cell apoptosis were the sum of those which were derived from early apoptosis and late apoptosis.

### RNA immunoprecipitation

When miR-126-3p mimics were transfected into HTR8/SVneo cells, RNA immunoprecipitation (RIP) was performed with the Magna RIP RNA-Binding Protein Immunoprecipitation Kit (EMD Millipore Corp., Billerica MA, USA) containing an AGO2-specific antibody (cell signaling technology), and an IgG antibody as a negative control. Harvested, the cells were centrifuged and re-suspended with 100 μL RIP lysis buffer supplemented with protease and RNase inhibitor. Mixed with antibody coupled beads, the lysate was kept in rotation at 4 °C overnight. Following proteinase K treatment, the immunoprecipitated RNAs were extracted and reversely transcribed as described in the section of RNA extraction and qRT-PCR. Subsequently, the levels of hsa_circ_0002348 were detected by qRT-PCR.

### Luciferase reporter assay

The wide-type and mutant fragments of *BAK1* 3′UTR cDNA were amplified by PCR. As indicated in Additional file 2: Table S2, a list was made of the cloning primers. The amplified products were synthesized and inserted into psiCHECK-2 vectors (Promega, Shanghai, China). Afterwards, the psiCHECK-2 vectors, miR-126-3p mimics and NC mimics were transfected, as the negative controls, into T293 cells using Lipofectamine 2000 (Invitrogen, USA). After 48 h-incubation, the luciferase activity was quantified with the Dual Luciferase Reporter Assay kit according to the manufacturer’s instructions (Promega, Shanghai, China).

### Western blot

When the cells were lysed, total protein was extracted with Radio Immunoprecipitation Assay (RIPA) lysis buffer (Thermo Scientific, USA). The lysates were resolved with 10% sodium dodecyl sulfate-polyacrylamide gel electrophoresis (SDS-PAGE), before transferred to polyvinylidenefluoride (PVDF) membranes to be probed with antibodies directed against phosphorylated TOR (Ser2448) (p-mTOR), mTOR, phosphorylated ERK1/2 (T202/Y204) (p-ERK1/2), ERK1/2, phosphorylated p38 (T180/Y182) (p-p38), p38, phosphorylated JNK (T183/Y185) (p-JNK), JNK, BAK1 (Cell Signaling Technology, USA), and β-tubulin (Abcam, USA). Developed with chemiluminescent HRP substrate (Thermo Scientific, USA), the bands were visualized, the intensity of which was quantified based on ImageJ software. All results were normalized to β-tubulin, which was used as a loading control. A detailed description was made of the antibodies (Additional file 4: Table S4).

### Hsa_circ_0002348 vector construction

As previously reported [16], hsa_circ_0002348 vector was established with modified plasmid called 3D5 based on the pZW1 vector (Additional file 5: Fig. S1). The whole sequence of hsa_circ_0002348 (Additional file 6: Table S5) was inserted into the 3D5 vector following digestion with restriction enzymes XhoI and PacI using a seamless cloning assay, and the product was transformed into STBL3 competent cells. The recombinant vector sequences were validated by Sanger sequencing.

### Animal experiments

Institute of Cancer Research (ICR) pregnant mice aged 10 weeks and weighted around 40 g (Shanghai Slac Laboratory Animal Co. Ltd.), were recorded as embryonic day 0 (E 0) with a copulation plug. All animal experiments were performed in accordance with the guidelines issued by Fudan University for the care and use of laboratory animals. The mice were raised in a light-, temperature- and humidity-controlled environment with free access to standard rodent chow and tap water. As previously described [17, 18], we succeeded in establishing a mouse model by intraperitoneally (i.p.) injecting lipopolysaccharide (LPS) (Escherichia coli serotype 0111:B4, Sigma-Aldrich, USA) twice into the pregnant mice at a dose of 100 μg/kg bodyweight on GD 5 and GD 10, respectively. To evaluate the role of hsa_circ_0002348 in the in vivo development of preeclampsia, additionally, we injected LPS-induced preeclampsia mice with hsa_circ_0002348 plasmids (320 μg/kg bodyweight) or an equal amount of pZW1 as blank controls, using Entranster™-in vivo Transfection Reagent (Engreen, China) on GD 8, 12 and 14, respectively. Thereby, the mice were randomly divided into four experimental groups: the control, LPS, LPS+pZW1 and LPS+hsa_circ_0002348. The controls were injected with the same volume of saline on GD 5, 8, 10, 12 and 14 correspondingly. Besides the injections received on GD 5 and 10, LPS group mice were injected with the same dosage of saline on GD 8, 12 and 14. The animal experiments were independently repeated for three times.

### Measurements of blood pressure and urinary protein in the mice

To evaluate the specific symptoms of preeclampsia, blood pressure (BP) and urinary protein were examined. The indirect BP was measured in the conscious mice by tail cuff plethysmography of the Visitech System BP2000 (Apex, NC, USA). All mice were habituated for 3 days before the formal start of BP measurement, at least 5 consecutive measurements recorded on GD 5, 14 and 16, respectively. At the same time points, the mice in the metabolic cages underwent quantification of their urinary protein concentration of 24 h urine samples, which used a CBB protein test kit (SNM297, Baiaolaibo Technology, Beijing, China) according to the manufacturer’s instructions.

### Assessment of kidney and placenta morphology in the mice

The mice’s kidneys were fixed and embedded in paraffin before sliced into 3-μm-thick serial sections, which were to be stained with hematoxylin and eosin (HE) or periodic acid-Schiff (PAS) for the examination of glomerular morphology. The mice’s placentas were stained with HE or Masson’s trichrome (MTC) staining for the examination of placental morphology. MTC was used to visualize collagen deposition, which was stained blue, while the muscle and cytoplasm appeared red to pink.

### ELISA assay in the mice

In the mice’s plasma, the protein levels of placental growth factor (PIGF), soluble fms-like tyrosine kinase-1 (sFlt-1), and tumor necrosis factor-α (TNF-α) were measured with enzyme-linked immunosorbent assay (ELISA) kits (ab197748; Abcam for PIGF, MBS9301224; Enzo Life Sciences, Inc. for sFlt-1, EMC102a.48; NeoBioscience for TNF-α) according to the manufacturer’s protocols.

### TUNEL assay in the mice

Terminal deoxynucleotidyl transferase (TdT)-mediated-digoxigenin-11-dUTP nick end labeling (TUNEL) was used to detect DNA fragmentation in placenta tissues of pregnant mice from the four experimental groups according to the instructions of TUNEL kit (KGA7063, KeyGENBioTECH, Jiangsu, China).

### Fluorescence in situ hybridization and immunohistochemistry

The human and pregnant mouse derived placental tissues were fixed in 4% formaldehyde solution and embedded in paraffin before sectioned. Fluorescence in situ hybridization (FISH) was performed to examine the expression of miR-126-3p, as previously described [19]. The miR-126-3p probes (GenePharma, China) were generated based on the following sequence: 5′-FAM-CGCATTATTACTCACGGTACGA-FAM-3′ and the sequence was identical in humans and mice. The probes were stained green, and the nuclei were counterstained blue with DAPI. The slides were reviewed under Nikon Fluorescence Microscope. For immunohistochemistry, the slides were deparaffinized in xylene and rinsed with PBS, before 30 min-pretreatment in 3% H_2_O_2_ and 50% methanol to eliminate the endogenous peroxidase activity. After that, the slides were disposed with a heat-induced epitope retrieval (HIER) method for antigen repair, then washed with PBS thrice and incubated at room temperature for 30 min in PBS containing 1% BSA blocking buffer, followed by an addition of BAK1 antibody (Cell Signaling Technology, USA), before incubated overnight at 4 °C. The slides were then washed thrice in PBS before incubated for 1 h at RT with the goat anti-rabbit secondary antibody. When the slides were rinsed thrice in PBS again, the reaction was developed with peroxidase substrate diaminobenzidine. Under a microscope was observed hematoxylin counterstain.

### Statistical analysis

Analysis of variance (ANOVA) was used to compare the variables from more than two groups; the two-tailed Student’s *t*-tests, to make comparisons between two groups; Pearson’s correlation and linear regression, to examine the relationship between the expression level of hsa_circ_0002348, SBP and DBP and the level of proteinuria in the patients with preeclampsia; repeated measures ANOVA, to analyze time-course data; and GraphPad Prism ver. 9 software and SPSS ver. 22 software, to perform statistical analyses. A P-value of ≤ 0.05 was considered statistically significant, and the data were expressed as mean ± standard error of the mean (SEM).

## Results

### Hsa_circ_0002348 was identified and confirmed to be upregulated in preeclampsia placentas

**Fig. 1:**
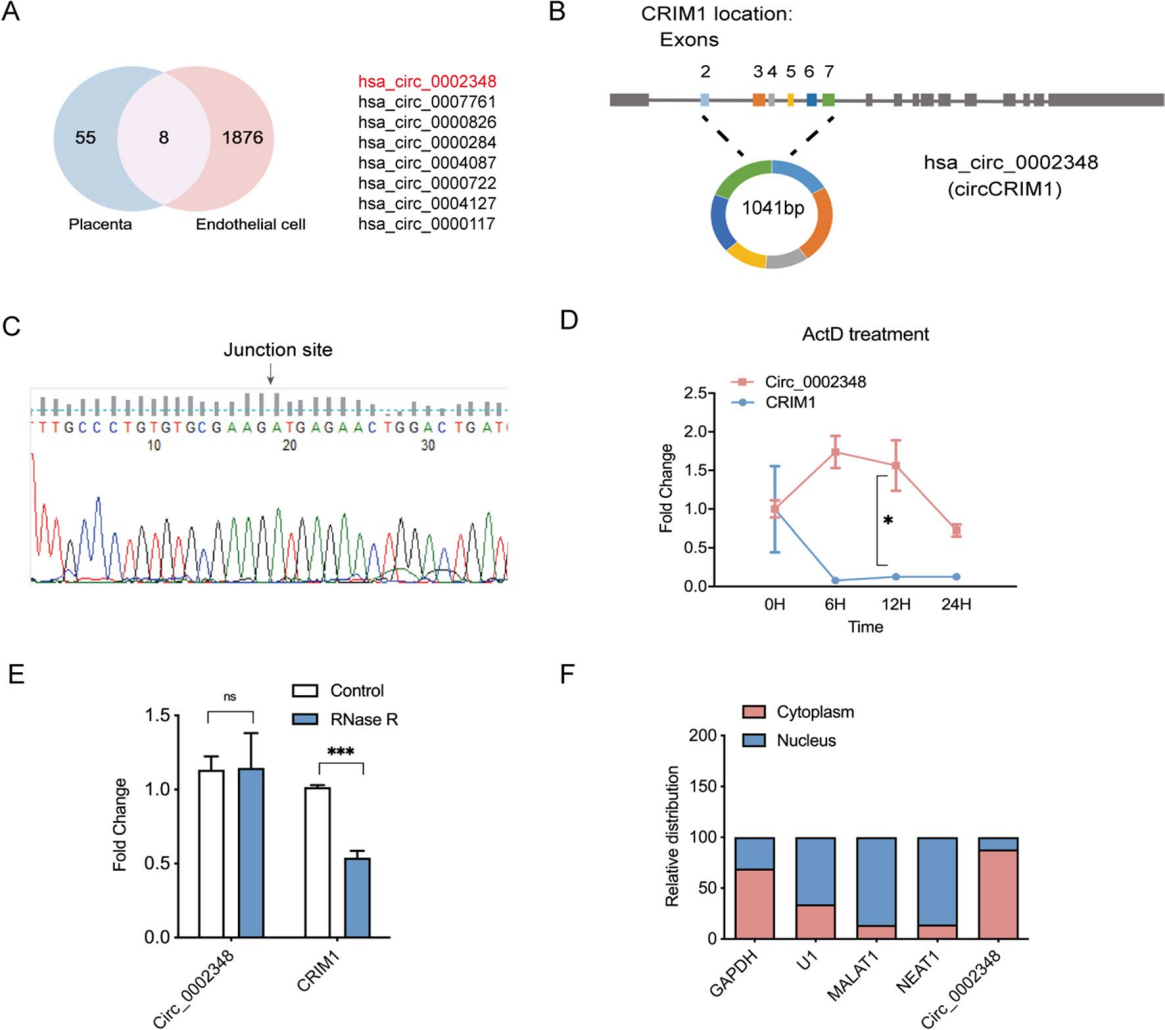
Identification and characteristics of hsa_circ_0002348. **A** Venn diagram showing overlapped circRNAs derived from both placental and endothelial cells; **B** schematic illustration showing the location of hsa_circ_0002348 in host gene *CRIM1*; **C** sanger sequencing validated the location of hsa_circ_0002348 in the *CRIM1* transcript; **D** qRT-PCR analysis detecting the stability of hsa_circ_0002348 in trophoblast cells after Act D treatment; **E** qRT-PCR analysis of hsa_circ_0002348, *CRIM1* expression after RNase R treatment in trophoblast cells; **F** relative expression of *GAPDH*, *U1*, *MALAT1*, *NEAT1*, hsa_circ_0002348 in the nucleus and cytoplasm, respectively; circ_0002348 representing hsa_circ_0002348

We analyzed the dataset GSE100242 in the GEO datasets [20], which presented a circRNA expression resource of 20 human tissues and 63 circRNAs were identified to be expressed in placenta tissue. Given that endothelial cells were also the main component of the placenta and endothelial dysfunction played a role in the development of preeclampsia [21], we examined a circRNA expression profile of human umbilical vein endothelial cells (HUVECs) containing 7388 circRNAs, of which 1884 had high-throughput sequencing of RNA isolated by cross-linking and immunoprecipitation (HITS-CLIP) peaks that determined a potential function as miRNA sponge [22]. Through intersection analysis of the host gene sequences of the 63 placenta and 1884 endothelial cells-derived circRNAs, it was found that the gene sequence of 9 circRNAs completely overlapped, of which 8 were annotated in circBase [23] and selected for further analysis. Considering that circRNAs and their cognate mRNAs may have similar functions in the same disease such as preeclampsia [24–26]. As a result, hsa_circ_0002348 became the research target for its host gene, CRIM1 expressed in both trophoblast and endothelial cells, and was essential for murine placenta development [27, 28] (Fig. 1A). Hsa_circ_0002348, located at chr2:36623756–36706837, consisted of 1041 nt, as the back-spliced circular product of the exon 2–7 of the CRIM1 transcript (Fig. 1B). The sequence of the splice junction of hsa_circ_0002348 was confirmed by Sanger sequencing, which was in concordance with its public sequence in circBase [23] (Fig. 1C). In order to further prove that hsa_circ_0002348 was a truly circRNA rather than a linear RNA, upon Act D treatment, the expression level of hsa_circ_0002348 was stable with a half-time > 24 h, while the linear transcript, CRIM1, was degraded with a half-time < 6 h (Fig. 1D) (p < 0.05). Additionally, hsa_circ_0002348 was resistant to the digestion of excessive RNase R with obviously degraded linear CRIM mRNAs, while the expression of the circular hsa_circ_0002348 remained unchanged (Fig. 1E) (p < 0.001). Furthermore, the separate qRT-PCR analysis of nuclear and cytoplasmic RNA exhibited that hsa_circ_0002348 was mainly present in the cytoplasm of HTR8/SVneo cells (Fig. 1F).

**Fig. 2:**
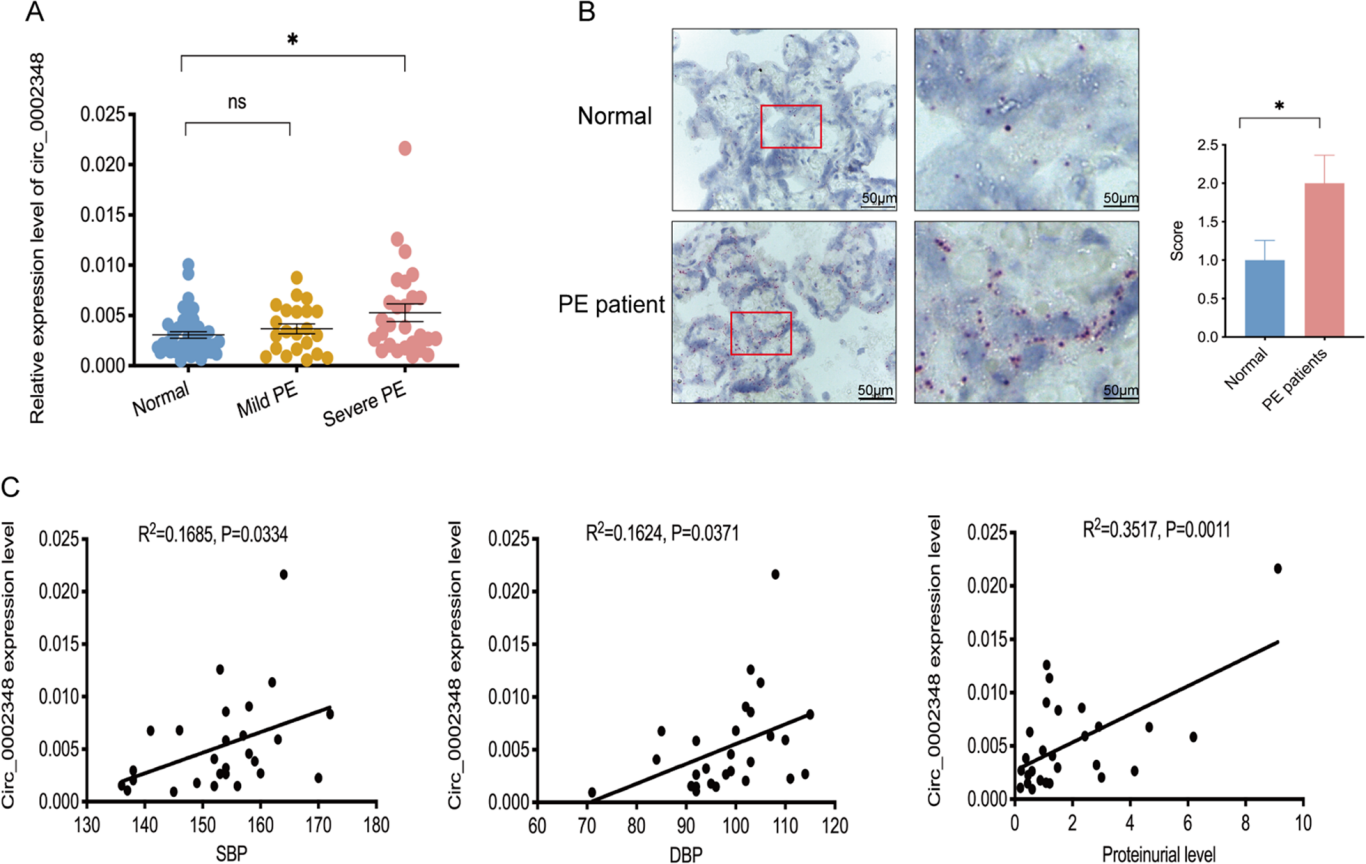
Hsa_circ_0002348 was upregulated in the placentas of preeclampsia patients. **A** qRT-PCR analysis of hsa_circ_0002348 expression in the placentas of preeclampsia patients with mild clinical symptoms (n = 22) and severe clinical symptoms (n = 27), compared with normal pregnant controls (n = 42); **B** representative images of BASEscope in situ hybridization (ISH) showing hsa_circ_0002348 expression in the placentas of preeclampsia patients (n = 3) and normal pregnant controls (n = 3); Scale bar, 50 μm; **C** correlation analysis of hsa_circ_0002348 expression and SBP, DBP, proteinuria level in preeclampsia patients; circ_0002348 representing hsa_circ_0002348; the data presented as mean ± SEM; *p ≤ 0.05, **p ≤ 0.01, ***p ≤ 0.001; *ns* not significant, *SBP* systolic blood pressure, *DBP* diastolic blood pressure

To identify whether hsa_circ_0002348 is associated with preeclampsia pathogenesis, we verified that it was significantly upregulated in placental tissues of 27 patients with severe preeclampsia, although not significant for those with mild clinical manifestation (n = 22), when compared with 22 normal controls (Fig. 2A) (p < 0.05). A significantly stronger positive signal of hsa_circ_0002348 was also observed in placental tissues of severe preeclampsia patients than in the controls of normal pregnancy (Fig. 2B) (p < 0.05). Furthermore, the expression of hsa_circ_0002348 was positively correlated with the systolic and diastolic BP and level of proteinuria in the patients with severe clinical manifestation (Fig. 2C) (p < 0.05), while no correlation was identified in the patients with mild clinical manifestation (Additional file 7: Fig. S2). These results showed that hsa_circ_0002348 was upregulated in preeclampsia placentas, indicating its involvement in the pathogenesis of preeclampsia.

### Hsa_circ_0002348 exacerbated preeclampsia-like symptoms in mice

**Fig. 3:**
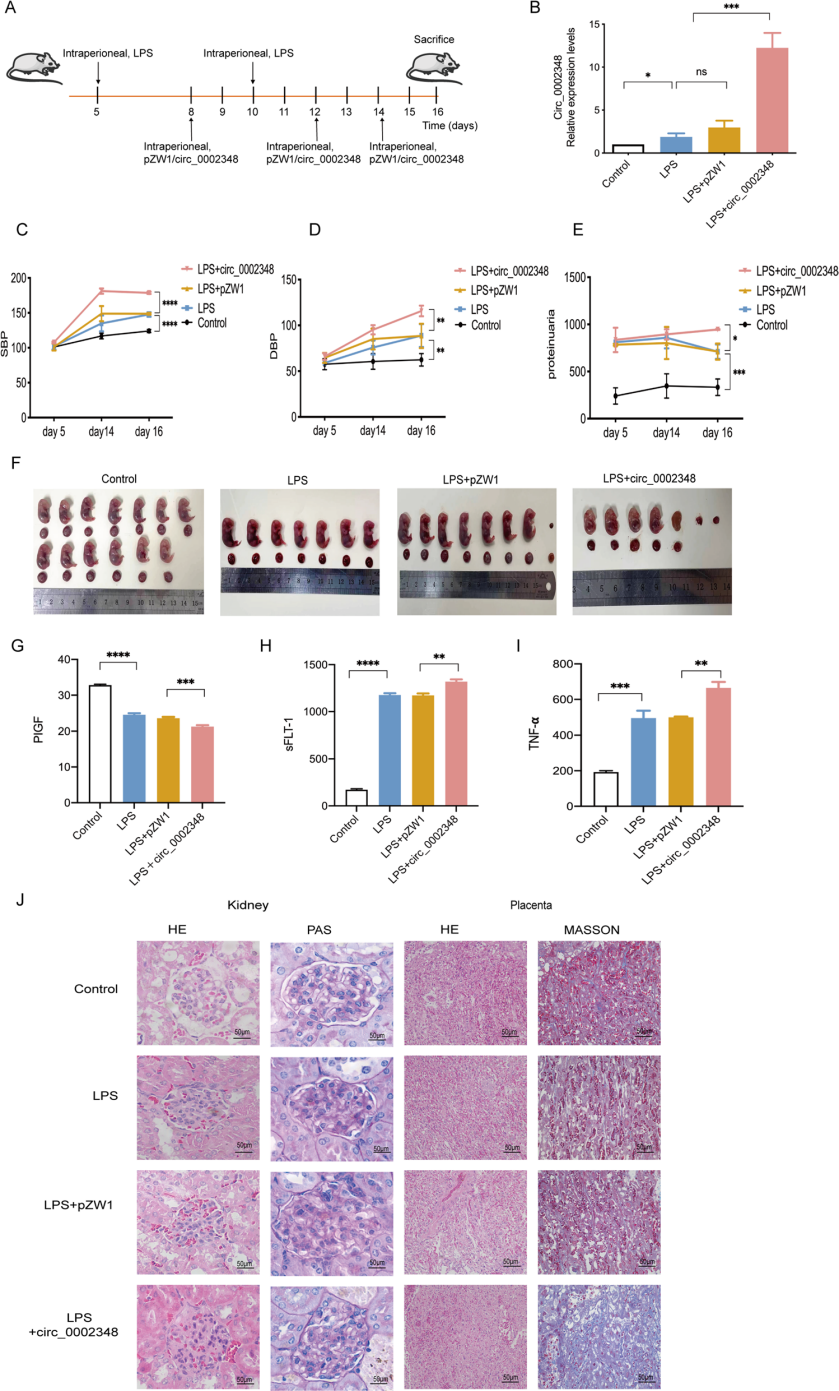
Hsa_circ_0002348 exacerbated preeclampsia-like symptoms in the mice. **A** Modeling schema of LPS-induced preeclampsia mice model, and treatment with hsa_circ_0002348 or pZW1 control vector (n = 3/group for each experiment); animal experiments independently repeated in triplicate; **B** qRT-PCR analysis of hsa_circ_0002348 in the mice’s placentas of each group; systolic BP **C**, diastolic BP **D**, and proteinuria level **E**, observed on GD 5, GD 14 and GD 16 in each group; **F** representative image of the fetal mice and placenta in the blank control, LPS, LPS+pZW1, and LPS+circ_0002348 group, respectively; ELISA analysis for PIGF detection (**G**), sFlt-1 **H** and TNF-α (**I**) in the plasmas of the pregnant mice of each group; **J** representative HE and PAS staining of the images in the pregnant mice’s kidneys, and representative HE and MASSON staining of the images in the pregnant mice’s placentas of each group; glomerular endotheliosis and abnormal stromal collagen deposition observed to be evident in the kidney and placental labyrinth layer, respectively; scar bars, 50 μm. circ_0002348 representing hsa_circ_0002348; the data presented as mean ± SEM; *p ≤ 0.05, **p ≤ 0.01, ***p ≤ 0.001, ****p ≤ 0.0001; *ns* not significant

To further figure out the cause-and-effect relationship between hsa_circ_0002348 and preeclampsia, we established a mouse model of preeclampsia using intraperitoneal (i.p.) injection of LPS and determined whether hsa_circ_0002348 overexpression vector could cause aggravation of preeclampsia (Fig. 3A). The successful induction of preeclampsia was confirmed, as manifested by the significantly higher systolic (Fig. 3C) (p < 0.0001) and diastolic BP (Fig. 3D) (p < 0.01) and proteinuria level (Fig. 3E) (p < 0.05), as well as by the reduced number of fetal mice and placentas (Fig. 3F), after LPS injections on GD 5 and GD 10. In line with results of human preeclampsia placentas, hsa_circ_0002348 was highly expressed in placentas of LPS-induced mouse model of preeclampsia (Fig. 3B). When concurrently injected with hsa_circ_0002348 vector on GD 8, GD 12 and GD 14, the mice displayed significantly enhanced expression of hsa_circ_0002348 in their placentas (Fig. 3B) (p < 0.05), afflicted with more severe preeclampsia-like symptoms such as fetal reabsorption besides higher BP, proteinuria, less fetal mice and placentas for LPS+circ_0002348 group when compared with LPS+pZW1-treated group (Fig. 3C–F) (p < 0.01). Compared with the controls, pregnant mice in LPS group displayed a significant decrease in PIGF expression (Fig. 3G) (p < 0.0001) and increase in sFlt-1 (Fig. 3H) (p < 0.0001), TNF-α (Fig. 3I) (p < 0.001), while more significant changing trends were observed in LPS+circ_0002348 group than in LPS+pZW1 group (Fig. 3G–I) (p < 0.01). Furthermore, the morphological changes in maternal kidney and placenta were assessed. The renal tissues showed relatively atrophic glomerular, and narrowed glomerular cystic cavities in pregnant mice after treated with LPS, LPS and hsa_circ_0002348 overexpression plasmid. Likewise, the labyrinth layer of placental tissues exhibited more blue staining in MTC staining, indicated a significant increase of stromal collagen deposition in LPS and LPS+circ_0002348 group when compared with the other three groups (Fig. 3J). Altogether, these findings revealed that hsa_circ_0002348 aggravated the typical clinical features and organ injury in the mouse model of LPS-induced preeclampsia.

### Hsa_circ_0002348 attenuated trophoblast proliferation and enhanced apoptosis

**Fig. 4:**
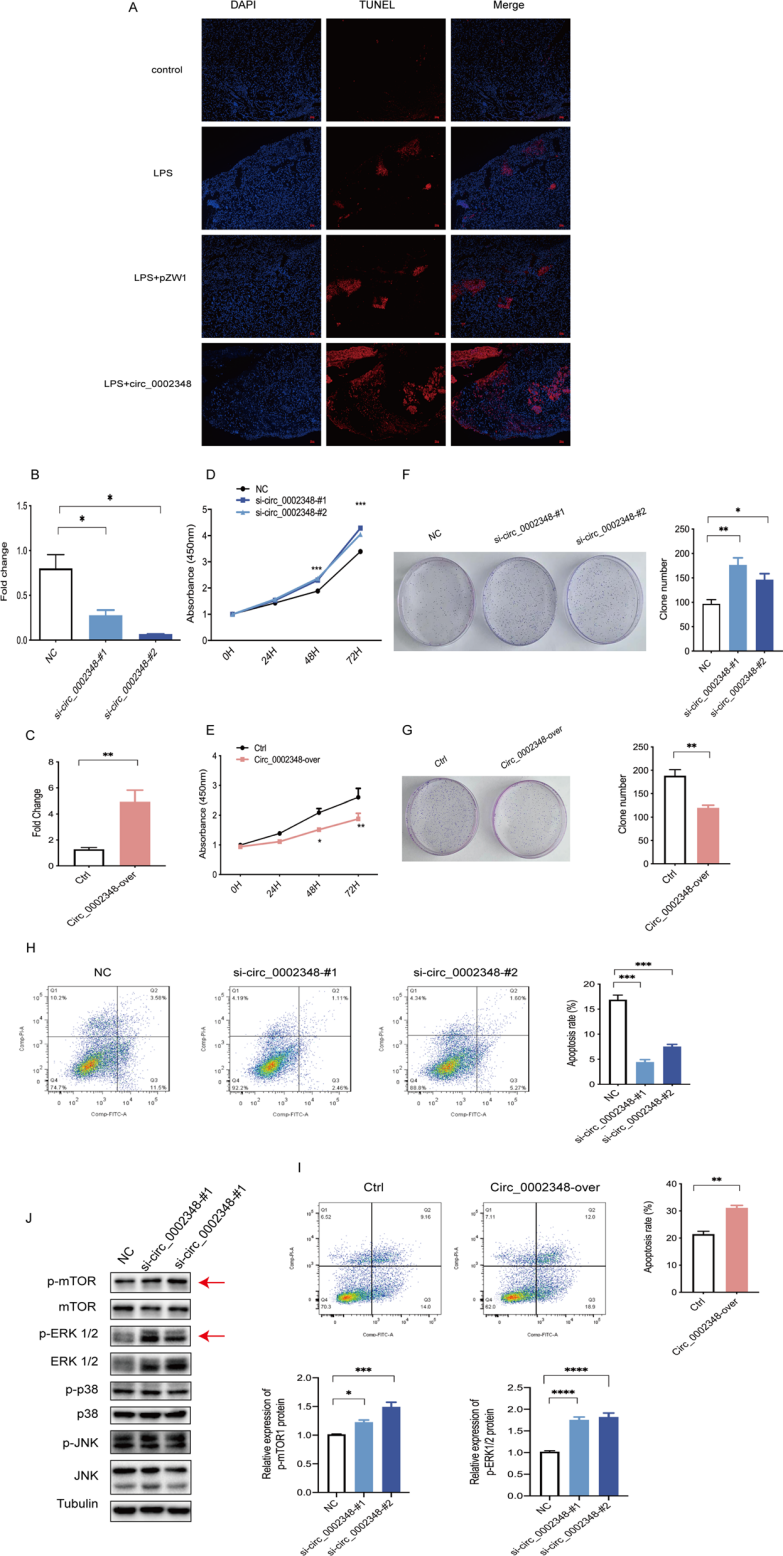
Hsa_circ_0002348 inhibited trophoblast proliferation and induced apoptosis through the repression of mTOR/ERK1/2 signaling pathway. **A** Representative images of TUNEL staining in the pregnant mice’s placentas of each group (n = 3/per group for each experiment); TUNEL signals in red, and DAPI-stained nuclei in blue; Scar bars, 50 μm; **B** qRT-PCR analysis of the expression levels of hsa_circ_0002348 in HTR8 cells following its knockdown by two distinct siRNAs; **C** qRT-PCR analysis of the expression levels of hsa_circ_0002348 in HTR8 cells with its overexpression vector; CCK-8 assay showing the proliferation ability of HTR8 cells after transfected with two siRNAs **D** and its overexpression vector **E**; colony-formation assays showing the proliferation capacity of HTR8 cells with two siRNAs **F** and hsa_circ_0002348 overexpression vector **G**; flow cytometry of fluorescence analysis stained with Annexin V-FITC and PI in HTR8 cells after hsa_circ_0002348 knockdown by two distinct siRNAs **H** and its overexpression vector **I**; the percentage of cells in the four different quadrants calculated, the results presented in different histograms indicating the fraction of the apoptotic cells as Annexin V^+^/PI^−^ and Annexin V^+^/PI^+^; the quantitative results of **F**–**I** displayed in the right; **J** western blotting analysis detecting the expression level of p-mTOR, mTOR, p-ERK1/2, ERK1/2, p-p38, p38, p-JNK and JNK when compared with Tubulin as control in HTR8 cells with NC or with two hsa_circ_0002348 siRNAs as treatment; the quantitative analysis of band intensity performed with Image J (right); circ_0002348 representing hsa_circ_0002348; *NC* negative control, *Ctrl* control; the data presented as mean ± SEM; *p ≤ 0.05, **p ≤ 0.01, ***p ≤ 0.001,****p ≤ 0.0001

Although trophoblast invasion is an initiating factor in the development of preeclampsia [29], apoptosis is particularly important to all stages of placental development and an activation of pro-apoptosis responses in the trophoblast cells could contribute to abnormal placentation in preeclampsia [6, 30]. Therefore, our focus was on trophoblast apoptosis. We investigated the effect of hsa_circ_0002348 on the apoptosis viability of placental trophoblast in the mouse model of LPS-induced preeclampsia. As indicated by EDU (red)/DAPI (blue) immunostaining assay, the apoptosis of trophoblast cells increased in the groups of LPS and LPS+pZW1, and increased more significantly in the group of LPS+circ_0002348 than in the control group (Fig. 4A).

Moreover, we explored the effect of hsa_circ_0002348 on the proliferation and apoptosis in vitro. We knocked down the expression of hsa_circ_0002348 using two small interfering RNAs (siRNAs) targeting the hsa_circ_0002348 junction site, and upregulated its expression by transfecting its overexpression vector. The knockdown and overexpression efficiency were confirmed by qRT-PCR (Fig. 4B, C) (p < 0.05). As shown by the CCK-8 assay, HTR8/SVneo proliferative ability was increased after transfection with si-circ_0002348-#1 and si-circ_0002348-#2 (Fig. 4D) (p < 0.001), but decreased with the hsa_circ_0002348 overexpression vector transfection (Fig. 4E) (p < 0.05). As verified by the colony formation assay, furthermore, HTR-8/SVneo cells proliferation was significantly promoted by the knockout of hsa_circ_0002348 (Fig. 4F) (p < 0.05), but inhibited by its overexpression (Fig. 4G) (p < 0.01). Additionally, when the cell apoptosis rate was tested by flow cytometry so as to evaluate the effect of hsa_circ_0002348 on trophoblast apoptosis, we found that the downregulation of hsa_circ_0002348 suppressed apoptosis significantly (Fig. 4H) (p < 0.001), whereas its upregulation promoted the total rate of apoptosis (Fig. 4I) (p < 0.01).

The mTOR and MAPK signaling pathways, which are both important intracellular signaling pathways that play a key role in regulating various cellular process such as cell proliferation and apoptosis [31, 32], are promising to be therapeutic targets [33]. Moreover, there is evidence that mTOR and MAPK pathways are involved in trophoblast biological activity in placenta-mediated pregnancy complications such as fetal growth restriction and preeclampsia [34, 35]. To investigate the possible pathways involved in hsa_circ_0002348 mediated trophoblast proliferation and apoptosis, we confirmed its role in mTOR and ERK1/2 pathways via western blot. Increased phosphorylation levels of mTOR and ERK1/2 were observed after hsa_circ_0002348 knockout in HTR8/SVneo cells. However, no apparent change was observed in the expression levels of p38 and JNK and their phosphorylation (Fig. 4J) (p < 0.05). Taken together, these results demonstrated that hsa_circ_0002348 regulated trophoblast proliferation and apoptosis through the repression of the mTOR and ERK1/2 pathway.

### Hsa_circ_0002348 regulated trophoblast proliferation and apoptosis by competitively binding to miR-126-3p

**Fig. 5:**
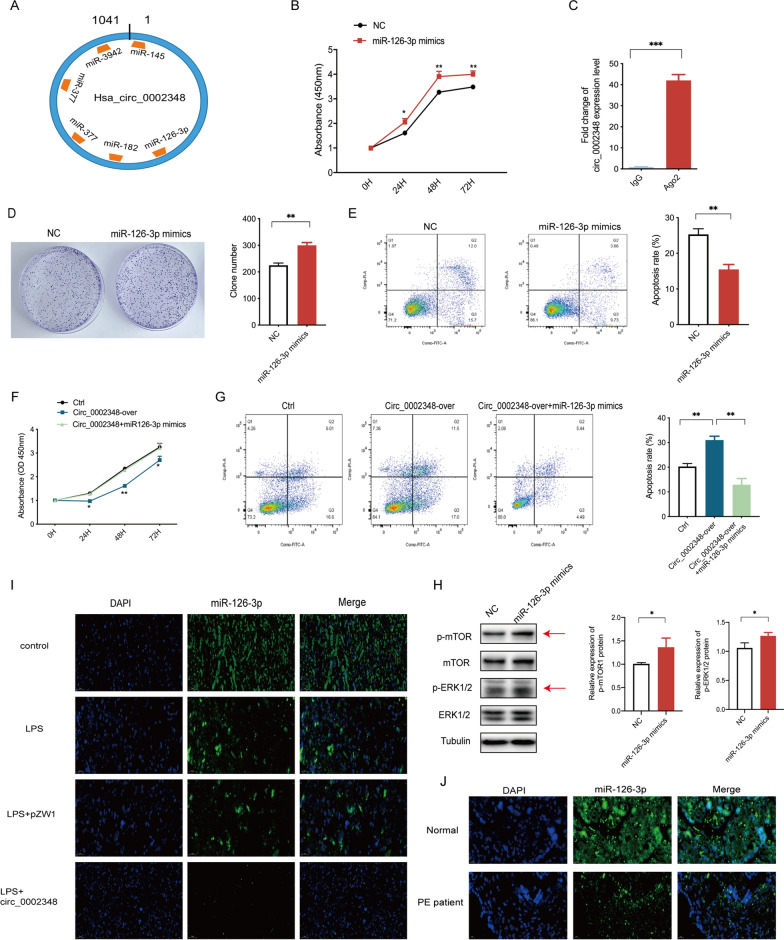
Hsa_circ_0002348 regulated trophoblast proliferation and apoptosis by competitively binding to miR-126-3p. **A** Schematic illustration showing hsa_circ_0002348 binding to five miRNAs predicted by online tools; **B** CCK-8 assay displaying the augmented trophoblast proliferation after transfected with miR-126-3p mimics; **C** RIP assay detected by qRT-PCR showing the enrichment of hsa_circ_0002348 pulled down by AGO2 in miR-126-3p mimics treated with HTR8 cells; **D** colony-formation assays showing the proliferation ability of HTR8 cells treated with miR-126-3p mimics; **E** flow cytometry of fluorescence analysis stained with Annexin V-FITC and PI detecting the apoptosis rate of HTR8 cells treated with miR-126-3p mimics; **F** CCK-8 assay showing cell proliferation of HTR8 cells with hsa_circ_0002348 overexpression and miR-126-3p mimics; **G** flow cytometry of fluorescence analysis stained with Annexin V-FITC and PI detecting the apoptosis rate of HTR8 cells treated with hsa_circ_0002348 overexpression and miR-126-3p mimics; the quantitative histograms of **D**, **E**, and **G** displayed in the right; **H** western blotting analysis showing the expression level of p-mTOR, mTOR, p-ERK1/2, ERK1/2 when compared with Tubulin as control in HTR8 cells with NC or miR-126-3p mimics; the quantitative analysis of band intensity performed with Image J (right); **I** representative FISH images of miR-126-3p expression in the pregnant mice’s placentas of each group (n = 3/group); **J** representative FISH images of miR-126-3p expression in the preeclampsia patients’ placentas (n = 3)and normal healthy controls (n = 3); miR-126-3p signals in green, and DAPI-stained nuclei in blue; Scar bars, 50 μm. circ_0002348 over representing hsa_circ_0002348 overexpression; *NC* negative control, *Ctrl* control; the data presented as mean ± SEM; *p ≤ 0.05, **p ≤ 0.01, ***p ≤ 0.001

As indicated by the subcellular fractionation and localization assays, hsa_circ_0002348 was primarily localized to the cytoplasm, which indicated that it most probably played a regulatory role in a post-transcriptional manner. Given that circRNAs function as miRNA sponge that protects mRNAs by competing for their targeting miRNAs, we hypothesized that it could play a similar role. Therefore, we searched for putative miRNAs binding to hsa_circ_0002348 by making bioinformatics analyses based on Circnet, Interactome and RNA22 software. Consequently, we obtained five miRNAs including miR-126-3p, miR-145, miR-182, miR-377 and miR-3942, all of which had potential binding sites with hsa_circ_0002348 (Fig. 5A). Moreover, when HTR8/SVneo cells were transfected with the mimics of all the five miRNAs, trophoblast proliferation capacity was markedly augmented (Additional file 8: Fig. S3), especially the miR-126-3p presenting the most significantly changing trends (Fig. 5B) (p < 0.05), revealed by CCK-8 assays. Thus, we focused on the miR-126-3p and explored its interaction with hsa_circ_0002348 using anti-AGO2 RIP in HTR8/SVneo cells to transiently upregulate miR-126-3p. When pulled down by AGO2, the endogenous hsa_circ_0002348 was enriched in the cells treated with miR-126-3p (Fig. 5C) (p < 0.001), which indicated that miR-126-3p targeted hsa_circ_0002348 directly. As shown by the colony formation assays, furthermore, the ectopic overexpression of miR-126-3p prominently promoted trophoblast proliferation (Fig. 5D) (p < 0.01). Under the same treatment, an obviously decreased rate of apoptosis was observed by flow cytometry assay (Fig. 5E) (p < 0.01).

Next, to determine whether hsa_circ_0002348 regulated trophoblast proliferation and apoptosis by competing for miR-126-3p, we conducted rescue assays. Ectopic expression of hsa_circ_0002348 was found to attenuate remarkably the trophoblast proliferation, while the overexpression of miR-126-3p reduced this attenuation (Fig. 5F) (p < 0.05). Meanwhile, the overexpression of hsa_circ_0002348 induced cell apoptosis dramatically, and the re-expression of miR-126-3p was capable of approximately reversing the effect of hsa_circ_0002348 on the cell apoptosis in trophoblast cells (Fig. 5G) (p < 0.01). Furthermore, western blot showed that the overexpression of miR-126-3p resulted in a slightly increased level of phosphorylated mTOR and ERK1/2 in trophoblast cells (Fig. 5H) (p < 0.05).

Additionally, FISH analysis validated the expression alteration of miR-126-3p in the mice with LPS-induced preeclampsia as well as in the patients with preeclampsia, showing that miR-126-3p was sharply decreased in both (Fig. 5I, J). Particularly, after the injection of hsa_circ_0002348 overexpression plasmid, the lower expression level of miR-126-3p was detected in the placentas of LPS+circ_0002348 group than in those of other three groups (Fig. 5I). These findings illustrated that hsa_circ_0002348 exerted its effect on trophoblast proliferation and apoptosis when interacting with miR-126-3p.

### Hsa_circ_0002348 regulated BAK1 expression by competing for miR-126-3p

**Fig. 6:**
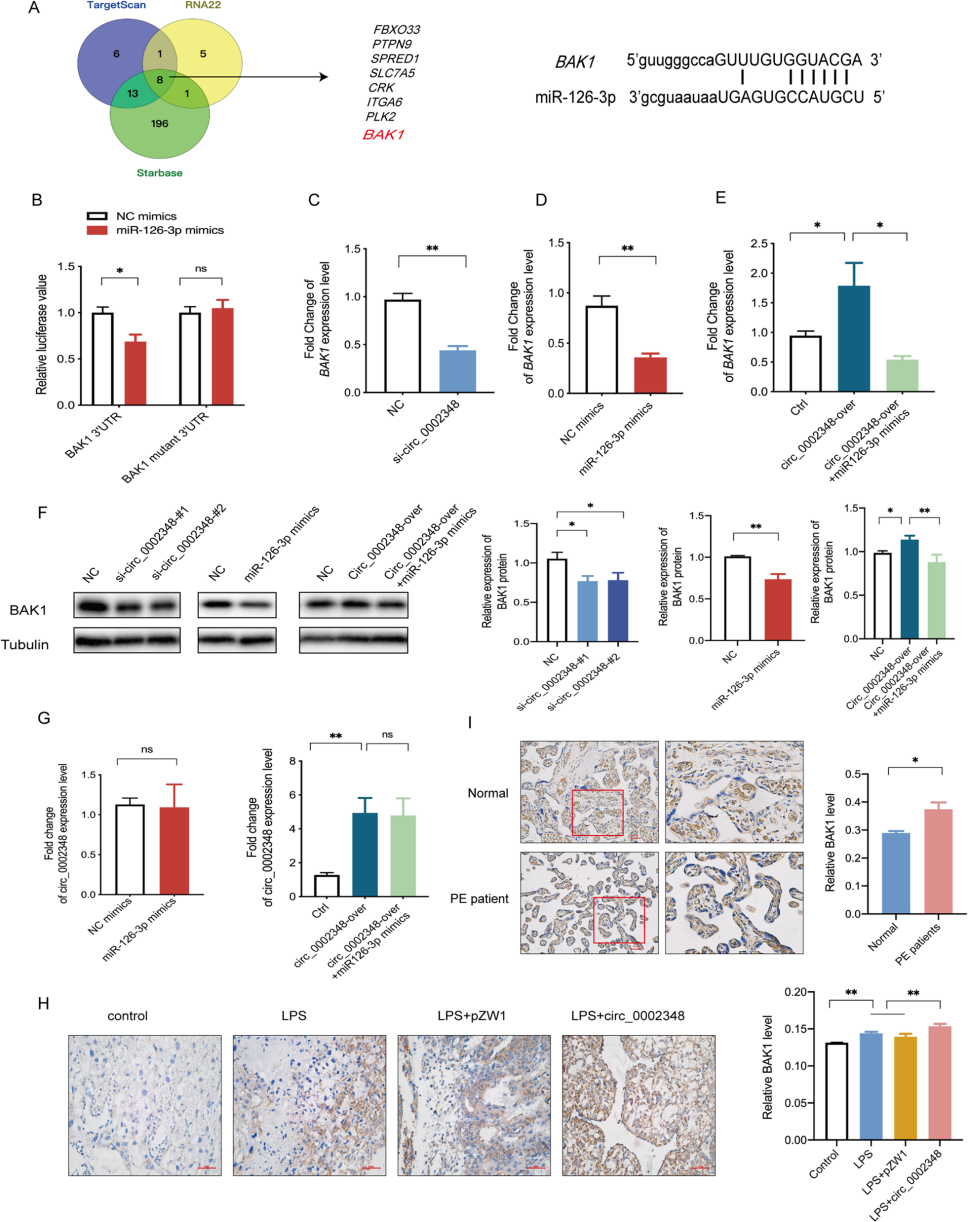
Hsa_circ_0002348 regulated BAK1 expression by competing for miR-126-3p. **A** Venn diagram showing the identification of miR-126-3p targeted genes, predicted from the public databases; the binding sites of miR-126-3p and BAK1 in the right; **B** luciferase reporter activity of *BAK1* 3′UTR in HTR8 cells co-transfected with miR-126-3p mimics and NC mimics; **C** qRT-PCR analysis of *BAK1* expression levels in HTR8 cells transfected with hsa_circ_0002348 knockdown; **D** qRT-PCR analysis of *BAK1* expression levels in HTR8 cells transfected with miR-126-3p mimics; **E** qRT-PCR analysis of *BAK1* expression levels in HTR8 cells treated with hsa_circ_0002348 overexpression and miR-126-3p mimics; **F** western blotting analysis showing the expression of BAK1 compared with Tubulin as internal control; the quantitative analysis of band intensity performed with Image J (right); **G** qRT-PCR analysis of hsa_circ_0002348 overexpression in HTR8 cells transfected with miR-126-3p mimics (left) and hsa_circ_0002348 overexpression co-transfected with or without miR-126-3p mimics (right); **H** representative IHC image of BAK1 expression in the pregnant mice’s placentas of each group (n = 3/group; left); the semi-quantitative analysis of BAK1 staining performed with Image J (right); **I** representative IHC image of BAK1 in the placentas of the preeclampsia patients (n = 3) and normal healthy controls (n = 3; left); the quantitative analysis performed with Image J (right); *NC* negative control, *Ctrl* control; circ_0002348 representing hsa_circ_0002348; circ_0002348 over representing hsa_circ_0002348 overexpression; the data presented as mean ± SEM; *p ≤ 0.05, **p ≤ 0.01, ***p ≤ 0.001

In the identification of the hsa_circ_0002348/miR-126-3p-regulated downstream target genes which might play a role in trophoblast proliferation and apoptosis of preeclampsia, an integrative analysis of three public databases including Starbase, TargetScan and RNA22, predicted eight common target genes (Fig. 6A), one of which as BAK1 of proapoptotic protein (member of Bcl-2 family), was of particular interest to us for its extensive expression in essentially all organs and its role as a regulator of apoptosis in multiple cell types [36]. Thus, we selected BAK1 for luciferase reporter experiment, where HTR8/SVneo cells were transfected with BAK1 3′UTR or BAK1 mutant 3′UTR fragments, before with miR-126-3p mimics or NC mimics. The results showed that the luciferase activity of BAK1 3′UTR decreased in the group of miR-126-3p mimics when compared with the group of NC mimics, which was not observed in BAK1 mutant 3′UTR (Fig. 6B) (p < 0.05). Next, to verify that BAK1 expression is indeed regulated through hsa_circ_0002348 and miR-126-3p, we conducted qRT-PCR and western blot analysis in HTR8/SVneo cells. Hsa_circ_0002348 silencing significantly decreased *BAK1* mRNA expression (Fig. 6C) (p < 0.01), so did miR-126-3p overexpression (Fig. 6D) (p < 0.01). Subsequent rescue experiments indicated that the overexpression of hsa_circ_0002348 significantly increased BAK1 expression, which was reversed by the co-expression of the miR-126-3p mimics (Fig. 5E) (p < 0.05). Similar results were validated at protein level by western blot analysis (Fig. 6F) (p < 0.05). However, miR-126-3p did not affect the expression of hsa_circ_0002348, even in the co-expression of hsa_circ_0002348 with miR-126-3p (Fig. 6G) (p < 0.01).

From IHC analysis conducted to further evaluate the effect of hsa_circ_0002348 on BAK1 protein expression in the placentas of the mice and patients, BAK1 protein expression was found to have increased in the placentas of the group of LPS and LPS+pZW1 when compared with the controls, with the highest level detected in the group of LPS+circ_0002348 (Fig. 6H) (p < 0.01). Similarly, BAK1 protein expression had a high level in the placentas of the patients (Fig. 6I) (p < 0.05). These data indicated that hsa_circ_0002348 played an important role in the placental pathogenesis of preeclampsia through miR-126-3p/BAK1 axis.

## Discussion

**Fig. 7:**
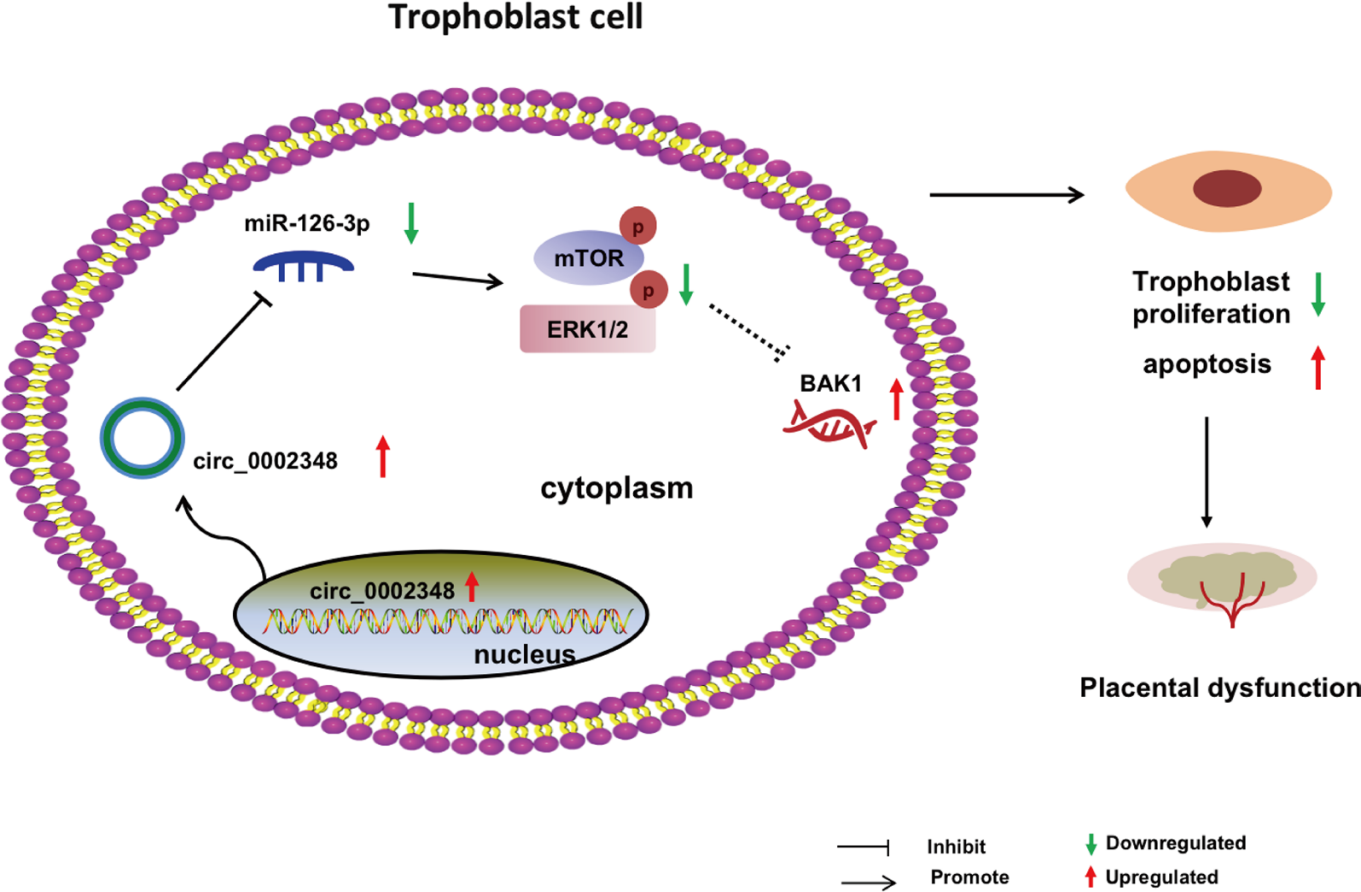
A proposed schematic for the function and regulatory mechanism of hsa_circ_0002348 in the pathogenesis of preeclampsia

A growing number of researches have revealed that numerous circRNAs function as a crucial regulator in quite a number of diseases, including preeclampsia. However, the distinctive role and regulatory mechanism of circRNAs remains poorly elucidated. In the present study, we determined a newly functional circRNA, namely hsa_circ_0002348 and revealed that it was highly expressed in preeclampsia placentas and correlated positively with clinical symptoms. Animal experiments showed that it exacerbated the preeclampsia-like symptoms of pregnant mice. Mechanistically, hsa_circ_0002348 inhibited trophoblast proliferation and promoted apoptosis through miR-126-3p/BAK1 ceRNA axis (Fig. 7). Therefore, we demonstrated that hsa_circ_0002348 had a new pathway governing the regulation of trophoblast proliferation and apoptosis in the pathogenesis of preeclampsia and could be a new potential therapeutic candidate for preeclampsia.

Recently, several circRNAs have been validated to serve as miRNA sponge or ceRNA to influence the expression of miRNA targets in various diseases [9, 37]. Likewise, in preeclampsia, an increasing number of studies have provided compelling evidence that some circRNAs from placentas could regulate different trophoblast functions, such as cell proliferation, migration, invasion, and apoptosis by binding to miRNAs, thus affecting the expression of downstream targeted genes [26, 38–48]. Consistent with these previous studies, in the current study, we demonstrated that hsa_circ_0002348 played an essential role in regulating proliferation and apoptosis, and further proved that hsa_circ_0002348/miR-126-3p/BAK1 ceRNA axis to trigger proapoptosis in the trophoblast cells. Different with most existing studies, and more importantly, we confirmed that miR-126-3p and BAK1 as the crucial components of ceRNA axis were dysregulated in the placentas of both mouse model of preeclampsia and human preeclampsia patients, which further supported the role of hsa_circ_0002348/miR-126-3p/BAK1 ceRNA axis in the placental pathophysiological process of preeclampsia. Although we performed the integrative bioinformatics analysis, cell and animal experiments to validate the role of hsa_circ_0002348/miR-126-3p/BAK1 ceRNA axis during trophoblast proliferation and apoptosis, we still need to pursue some other regulatory mechanisms such as RBPs or encoding proteins. Further research is needed to prove these possibilities.

Interestingly, miR-126-3p was initially proposed as an endothelial-specific miRNA [49] to modulate the expression of genes involved in angiogenic pathways such as vascular endothelial growth factor (*VEGF*) [50], besides, it also has anti-apoptotic and anti-inflammatory properties [51]. It was later reported that trophoblast cells expressed miR-126-3p as well, and their aberrant expression contributed to increased inflammatory response in the placentas of preeclampsia [52]. In contrast, in this study, we found that miR-126-3p expression was lower in preeclampsia placentas than normal controls and it could regulate the apoptosis ability together with hsa_circ_0002348 in trophoblast cells. Therefore, our study identified a new potential function of miR-126-3p in preeclampsia.

BAK1, one important member of BCL-2 family, can activate the intrinsic apoptosis pathway [53]. Moreover, it has been reported that the change of BAK expression was one of the factors regulating the apoptosis of human trophoblast cells [54], thus indirectly speculating its involvement in trophoblast-related disease such as preeclampsia [55]. However, few studies have been reported where BAK1 expression was directly detected in the placental tissue of preeclampsia. In the current study, we determined a change of enhanced BAK1 expression in placenta tissue from both preeclampsia women and LPS-induced preeclampsia mice model, showing a more definite association between BAK1 and preeclampsia. Additionally, little is known about the upstream regulatory networks of BAK1 in preeclampsia. Previous studies reported that BAK-dependent apoptotic activity could be enhanced by autophagy inhibition, which was closely related to mTOR pathway [56, 57]. Additionally, ERK1/2 signaling was confirmed to inhibit a classical BAK-dependent apoptosis induced by endoplasmic reticulum stress [58]. These studies have suggested that mTOR/ERK1/2 signaling can possibly regulate BAK1 expression. In the present study, we demonstrated BAK1 expression was regulated by hsa_circ_0002348/miR-126-3p axis and miR-126-3p overexpression had a positive effect on mTOR/ERK1/2 signaling in trophoblast cells. Therefore, we inferred that BAK1 might be involved in regulating trophoblast apoptosis of preeclampsia mediated by hsa_circ_0002348/miR-126-3p axis through mTOR/ERK1/2 signaling. Thus, further research is needed to verify the regulation role of the mTOR/ERK1/2 signaling/BAK1 axis in preeclampsia.

The preeclampsia animal model has been successfully constructed in rats or mice with LPS administration via i.p. [17] or tail injection [18]. LPS, a toxic component of the cell walls of Gram-negative bacteria, has been shown to impair spiral artery remodeling and alter uteroplacental perfusion, leading to abnormal maternal inflammation [17]. This model has proven not only to simulate the typical phenotypes and pathogenesis of preeclampsia, but to be of great use for investigating some new preventive and therapeutic approaches to this multi-factorial disease [59, 60]. Of note, we optimized the animal model for LPS exposure on GD 5 and GD 10, respectively, probably corresponding to the key time window of human placental formation [61], in which we focused the effect of hsa_circ_0002348 on the placenta development of LPS-induced preeclampsia. To the best of our knowledge, this is the first study to reveal the role that circRNA plays in preeclampsia through animal experiments. In accordance with our in vitro studies on the increased apoptosis of trophoblast cells, we demonstrated, to some extent, that hsa_circ_0002348 could mediate miR-126-3p downregulation and BAK1 upregulation, which could be associated with trophoblast apoptosis in the mouse model of LPS-induced preeclampsia.

In summary, based on the human sample data, cell experiment and animal experiment, it was concluded that we identified a new functional circRNA, termed hsa_circ_0002348, demonstrating an evidence that it could regulate trophoblast proliferation and apoptosis mediated via hsa_circ_0002348/miR-126-3p/BAK1 axis in preeclampsia. As a valuable insight into the pathophysiology of preeclampsia, these findings could potentially lead to new therapeutic strategies. However, there were several limitations in this study. We only measured the expression of hsa_circ_0002348 in placenta tissue; it is important to continue to validate its expression in maternal peripheral blood to make it more likely to be a biomarker for clinical transformation. Next, only the trophoblast proliferation and apoptosis were discovered; although these two functions are crucial for placenta development, the invasion and migration functions are also indispensable, and thus additional studies are needed. Additionally, given the diversity of circRNA functions, hsa_circ_0002348 could function by other downstream molecules and mechanisms besides ceRNA networks of hsa_circ_0002348/miR-126-3p/BAK1 axis. Further research is needed to gain a more comprehensive understanding of hsa_circ_0002348 in preeclampsia.

## Supplementary Information

**Additional file 1:** **Table S1.** Comparisons of the study population’s general characteristics among normal pregnancy, mild preeclampsia and severe preeclampsia.

**Additional file 2:** **Table S2.** Primer sequences used for amplification.

**Additional file 3:** **Table S3.** Sequences of NC and siRNAs.

**Additional file 4:** **Table S4.** All antibodies.

**Additional file 5:** **Figure S1.** Schematic map of the *3D5-circ_0002348* vector.

**Additional file 6:** **Table S5.** Sequences of hsa_circ_0002348.

**Additional file 7:** **Figure S2.** Correlation analysis of the expression of hsa_circ_0002348 and SBP, DBP, and proteinuria level in preeclampsia patients with mild clinical symptoms.

**Additional file 8:** **Figure S3.** CCK-8 analysis of trophoblast proliferation capacity after HTR8/SVneo cells were transfected with the mimics of all the five miRNAs predicted by online tools.

## Data Availability

All of the relevant data and materials are freely available to any investigator upon request.
