# Supplementary material for: Hsa_circ_0002348 regulates trophoblast proliferation and apoptosis through miR-126-3p/BAK1 axis in preeclampsia
Source: J Transl Med. 2023 Jul 28;21:509. doi: 10.1186/s12967-023-04240-1 (PMC10375637; doi:10.1186/s12967-023-04240-1)
Supplement: Supplementary file 1 — Additional file 1: Table S1. Comparisons of the study population’s general characteristics among normal pregnancy, mild preeclampsia and severe preeclampsia. [file 12967_2023_4240_MOESM1_ESM.docx]

Table S1 Comparisons of the study population’s general characteristics among normal pregnancy, mild preeclampsia and severe preeclampsia

|  | Normal pregnancy | Mild preeclampsia | Severe preeclampsia |
| --- | --- | --- | --- |
|  | n=42 | n=22 | n=27 |
| Maternal age (yrs) | 29.46 ± 2.64 | 30.75 ± 3.54 | 30.9 ± 3.92 |
| Body mass index (kg/m2) | 22.48 ± 1.93 | 24.92 ± 4.42 | 24.17 ± 4.3 |
| Gestation of delivery (wks) | 38.75 ± 0.62 | 38.39 ± 0.61 | 36.12 ± 2.0 ##✧ |
| Birth weight (g) | 3321.92 ± 345.16 | 3321.43 ± 484.68 | 2414.5 ± 659.42 ##✧ |
| SBP (mmHg) | 111.38 ± 13.27 | 143.43 ± 12.12** | 151.75 ± 10.69## |
| DBP (mmHg) | 71.65 ± 11.21 | 95.0±7.21** | 97.7 ± 10.87## |
| 24-hr proteinuria (g) | / | 0.65 ± 0.47 | 2.57 ± 2.28✧ |

Data presented as mean ± SEM

* &** = P<0.05 and P<0.001 for comparisons between mild preeclampsia and normal pregnancy, respectively

# &## =P<0.05 and P<0.001 for comparisons between severe preeclampsia and normal pregnancy, respectively

✧&✧✧=P<0.05 and P<0.001 for comparisons between severe and mild preeclampsia, respectively
