# Supplementary material for: Hsa_circ_0002348 regulates trophoblast proliferation and apoptosis through miR-126-3p/BAK1 axis in preeclampsia
Source: J Transl Med. 2023 Jul 28;21:509. doi: 10.1186/s12967-023-04240-1 (PMC10375637; doi:10.1186/s12967-023-04240-1)
Supplement: Supplementary file 2 — Additional file 2: Table S2. Primer sequences used for amplification. [file 12967_2023_4240_MOESM2_ESM.docx]

Table S2 Primer sequences used for amplification

| Name | Usage | Sequence (5’-3’) |
| --- | --- | --- |
| β-actin | qPCR forward | CACCATTGGCAATGAGCGGTTC |
|  | qPCR reverse | AGGTCTTTGCGGATGTCCACGT |
| GAPDH | qPCR forward | GTCTCCTCTGACTTCAACAGCG |
|  | qPCR reverse | ACCACCCTGTTGCTGTAGCCAA |
| U1 | qPCR forward | GGGAGATACCATGATCACGAAGGT |
|  | qPCR reverse | CCACAAATTATGCAGTCGAGTTTCCC |
| MALAT1 | qPCR forward | CTTAAGCGCAGCGCCATTTT |
|  | qPCR reverse | CCTCCAAACCCCAAGACCAA |
| NEAT1 | qPCR forward | GCTGGACCTTTCATGTAACGGG |
|  | qPCR reverse | TGAACTCTGCCGGTACAGGGAA |
| Hsa_circ_0002348 | qPCR forward | GTTGCCCTGTGTGCGAAGAT |
|  | qPCR reverse | ATTGCTGCAGGTTCGAATGGT |
| Hsa_circ_0000826 | qPCR forward | AGCGTCCAGTGGATGTAGCA |
|  | qPCR reverse | ACAGCTGTTCAGCTAGCTTCT |
| Hsa_circ_0007761 | qPCR forward | TGGAATCTGTGGGTTGAGGC |
|  | qPCR reverse | TTCTTTCCGCTCCTTCCCGA |
| CRIM1 | qPCR forward | ACGCGATCACAATGGTTGTCGG |
|  | qPCR reverse | GGCATCAGTAAGGAAACCGAAGG |
| miRNA126-3p mimics | Sense sequence | UCGUACCGUGAGUAAUAAUGCG |
| miRNA145-5p mimics | Sense sequence | GUCCAGUUUUCCCAGGAAUCCCU |
| miRNA182-5p mimics | Sense sequence | UUUGGCAAUGGUAGAACUCACACU |
| miRNA377-3p mimics | Sense sequence | AUCACACAAAGGCAACUUUUGU |
| miRNA3942 mimics | Sense sequence | AAGCAAUACUGUUACCUGAAAU |
| NC mimics | Sense sequence | UUCUCCGAACGUGUCACGUTT |
| *BAK1* | qPCR forward | TTACCGCCATCAGCAGGAACAG |
|  | qPCR reverse | GGAACTCTGAGTCATAGCGTCG |
| *BAK1*3’UTR | PCR forward | TACTCGAGTGGGACCTCCTTAGCCCTGTCTGCT |
|  | PCR reverse | AAGCGGCCGCCCCTGCATTTGGCTGAATCAAGAAC |
| *BAK1*mutant3’UTR | PCR forward | GCTGGTGGTAGACCCACACCTTCTGTTGGG |
|  | PCR reverse | CCCAACAGAAGGTGTGGGTCTACCACCAGC |
