# Supplementary material for: Hsa_circ_0002348 regulates trophoblast proliferation and apoptosis through miR-126-3p/BAK1 axis in preeclampsia
Source: J Transl Med. 2023 Jul 28;21:509. doi: 10.1186/s12967-023-04240-1 (PMC10375637; doi:10.1186/s12967-023-04240-1)
Supplement: Supplementary file 3 — Additional file 3: Table S3. Sequences of NC and siRNAs. [file 12967_2023_4240_MOESM3_ESM.docx]

Table S3 Sequences of NC and siRNAs

| Name | Target mRNA | Sense sequence (5’-3’) |
| --- | --- | --- |
| si-circ_0002348-#1 | Circ_0002348 | CCUGUGUGCGAAGAUGAGATT |
| si-circ_0002348-#2 | Circ_0002348 | GUGUGCGAAGAUGAGAACUTT |
| NC | -- | UUCUCCGAACGUGUCACGUTT |
|  |  |  |
