# Supplementary material for: Hsa_circ_0002348 regulates trophoblast proliferation and apoptosis through miR-126-3p/BAK1 axis in preeclampsia
Source: J Transl Med. 2023 Jul 28;21:509. doi: 10.1186/s12967-023-04240-1 (PMC10375637; doi:10.1186/s12967-023-04240-1)
Supplement: Supplementary file 4 — Additional file 4: Table S4. All antibodies. [file 12967_2023_4240_MOESM4_ESM.docx]

Table S4 All antibodies

| Description | Sources | Cat./clone | Dilutions/WB | Dilutions/IHC/FISH |
| --- | --- | --- | --- | --- |
| BAK1 for WB | CST, USA | 3814S | 1:1000 | / |
| BAK1 for IHC | CST, USA | 12105S | / | 1:400 |
| P38 | CST, USA | 9216S | 1:1000 | / |
| p-p38 | CST, USA | 9216S; 28B10 | 1:1000 | / |
| JNK | CST, USA | 9258S; 56G8 | 1:1000 | / |
| p-JNK | CST, USA | 9251S | 1:1000 | / |
| ERK1/2 | CST, USA | 4695S;137F5 | 1:1000 | / |
| p-ERK1/2 | CST, USA | 4376S; 20G11 | 1:1000 | / |
| mTOR | CST, USA | 2983S;7C16 | 1:1000 | / |
| p- mTOR | CST, USA | 5536S; D9C2 | 1:1000 | / |
| β-Tubulin | Abcam, Cambridge, UK | ab6046 | 1:5000 | / |
