# Supplementary figures and images for: Hsa_circ_0002348 regulates trophoblast proliferation and apoptosis through miR-126-3p/BAK1 axis in preeclampsia
Source: J Transl Med. 2023 Jul 28;21:509. doi: 10.1186/s12967-023-04240-1 (PMC10375637; doi:10.1186/s12967-023-04240-1)

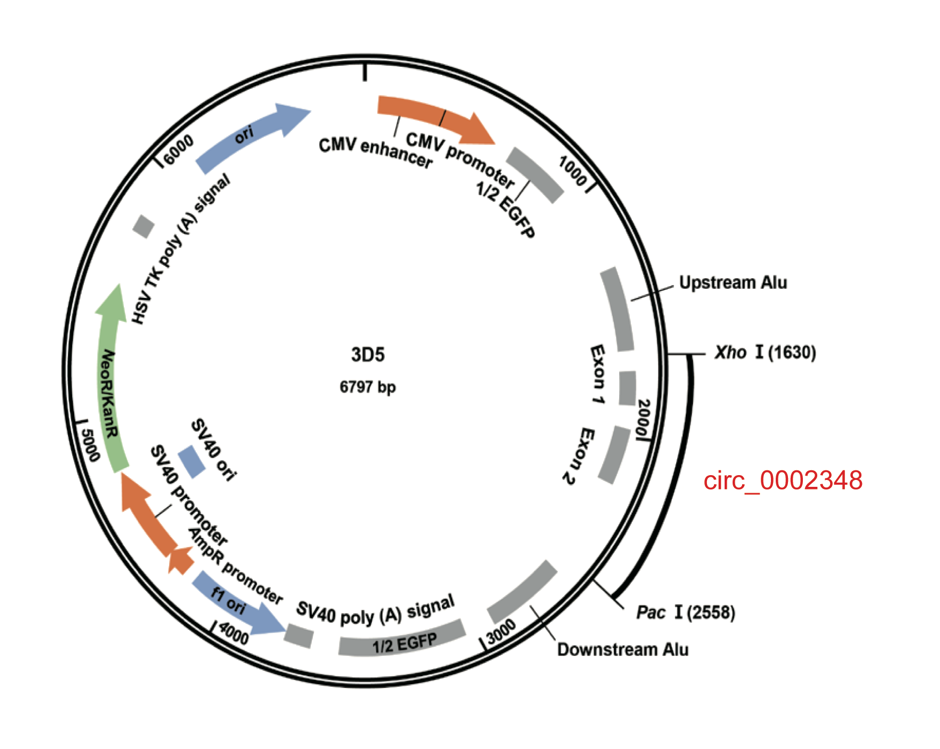


Figure S1 Schematic map of the *3D5-circ_0002348* vector

Supplement: Supplementary file 5 — Additional file 5: Figure S1. Schematic map of the 3D5-circ_0002348 vector. [file 12967_2023_4240_MOESM5_ESM.docx]
