# Supplementary material for: Hsa_circ_0002348 regulates trophoblast proliferation and apoptosis through miR-126-3p/BAK1 axis in preeclampsia
Source: J Transl Med. 2023 Jul 28;21:509. doi: 10.1186/s12967-023-04240-1 (PMC10375637; doi:10.1186/s12967-023-04240-1)
Supplement: Supplementary file 6 — Additional file 6: Table S5. Sequences of hsa_circ_0002348. [file 12967_2023_4240_MOESM6_ESM.docx]

Table S5 Sequences of hsa_circ_0002348

5‘

ATGAGAACTGGACTGATGACCAACTGCTTGGTTTTAAACCATGCAATGAAAACCTTATTGCTGGCTGCAATATAATCAATGGGAAATGTGAATGTAACACCATTCGAACCTGCAGCAATCCCTTTGAGTTTCCAAGTCAGGATATGTGCCTTTCAGCTTTAAAGAGAATTGAAGAAGAGAAGCCAGATTGCTCCAAGGCCCGCTGTGAAGTCCAGTTCTCTCCACGTTGTCCTGAAGATTCTGTTCTGATCGAGGGTTATGCTCCTCCTGGGGAGTGCTGTCCCTTACCCAGCCGCTGCGTGTGCAACCCCGCAGGCTGTCTGCGCAAAGTCTGCCAGCCGGGAAACCTGAACATACTAGTGTCAAAAGCCTCAGGGAAGCCGGGAGAGTGCTGTGACCTCTATGAGTGCAAACCAGTTTTCGGCGTGGACTGCAGGACTGTGGAATGCCCTCCTGTTCAGCAGACCGCGTGTCCCCCGGACAGCTATGAAACTCAAGTCAGACTAACTGCAGATGGTTGCTGTACTTTGCCAACAAGATGCGAGTGTCTCTCTGGCTTATGTGGTTTCCCCGTGTGTGAGGTGGGATCCACTCCCCGCATAGTCTCTCGTGGCGATGGGACACCTGGAAAGTGCTGTGATGTCTTTGAATGTGTTAATGATACAAAGCCAGCCTGCGTATTTAACAATGTGGAATATTATGATGGAGACATGTTTCGAATGGACAACTGTCGGTTCTGTCGATGCCAAGGGGGCGTTGCCATCTGCTTCACCGCCCAGTGTGGTGAGATAAACTGCGAGAGGTACTACGTGCCCGAAGGAGAGTGCTGCCCAGTGTGTGAAGATCCAGTGTATCCTTTTAATAATCCCGCTGGCTGCTATGCCAATGGCCTGATCCTTGCCCACGGAGACCGGTGGCGGGAAGACGACTGCACATTCTGCCAGTGCGTCAACGGTGAACGCCACTGCGTTGCGACCGTCTGCGGACAGACCTGCACAAACCCTGTGAAAGTGCCTGGGGAGTGTTGCCCTGTGTGCGAAG

3‘
