# Supplementary material for: Hsa_circ_0002348 regulates trophoblast proliferation and apoptosis through miR-126-3p/BAK1 axis in preeclampsia
Source: J Transl Med. 2023 Jul 28;21:509. doi: 10.1186/s12967-023-04240-1 (PMC10375637; doi:10.1186/s12967-023-04240-1)
Supplement: Supplementary file 7 — Additional file 7: Figure S2. Correlation analysis of the expression of hsa_circ_0002348 and SBP, DBP, and proteinuria level in preeclampsia patients with mild clinical symptoms. [file 12967_2023_4240_MOESM7_ESM.docx]

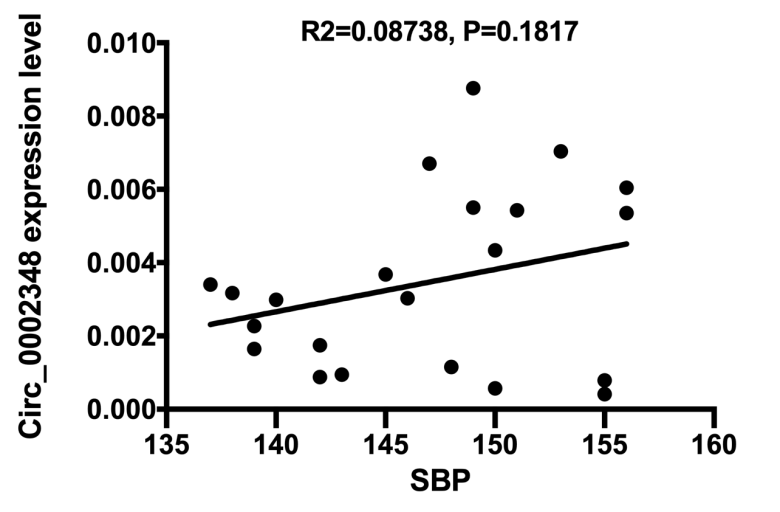

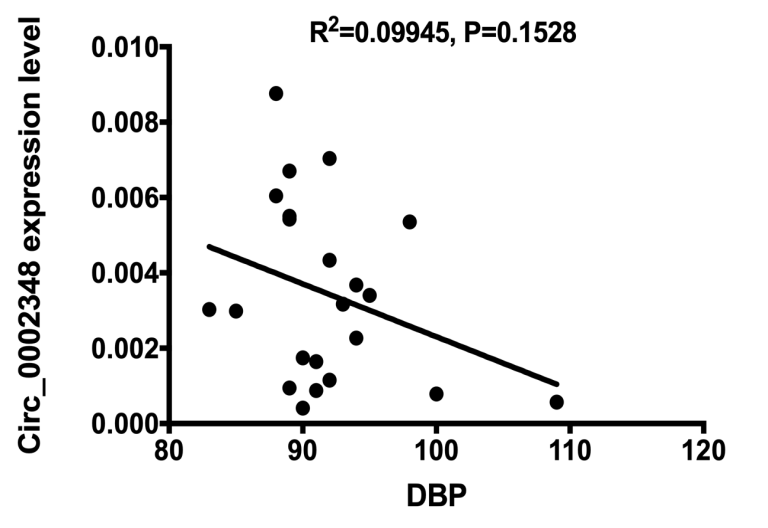

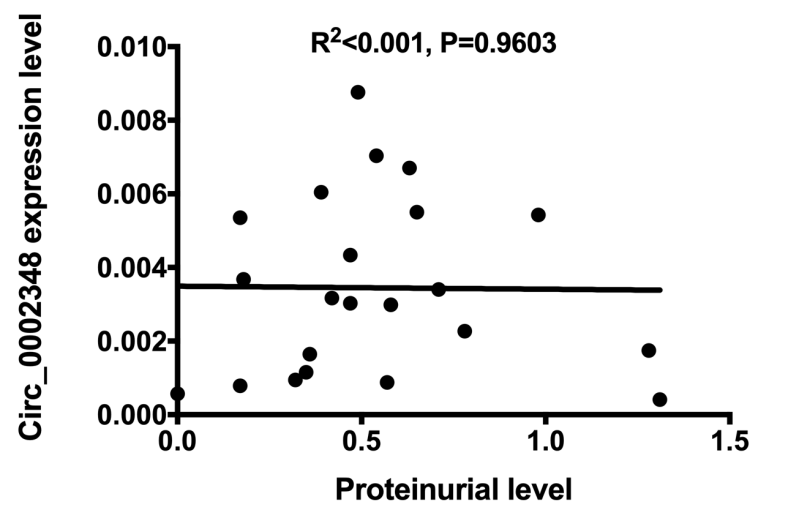


Figure S2 Correlation analysis of the expression of hsa_circ_0002348 and SBP, DBP, and proteinuria level in preeclampsia patients with mild clinical symptoms
