# Supplementary material for: Hsa_circ_0002348 regulates trophoblast proliferation and apoptosis through miR-126-3p/BAK1 axis in preeclampsia
Source: J Transl Med. 2023 Jul 28;21:509. doi: 10.1186/s12967-023-04240-1 (PMC10375637; doi:10.1186/s12967-023-04240-1)
Supplement: Supplementary file 8 — Additional file 8: Figure S3. CCK-8 analysis of trophoblast proliferation capacity after HTR8/SVneo cells were transfected with the mimics of all the five miRNAs predicted by online tools. [file 12967_2023_4240_MOESM8_ESM.docx]

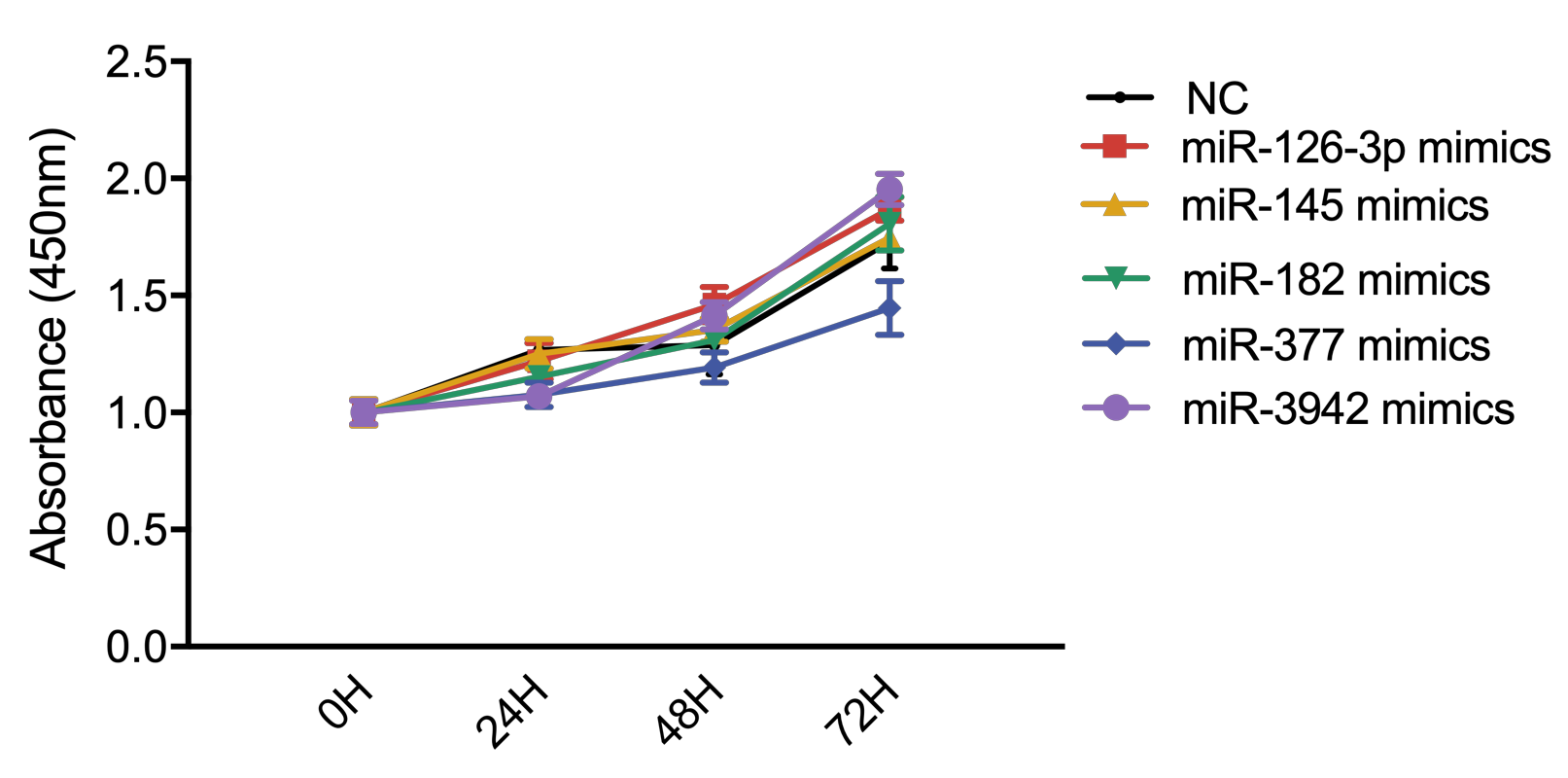


Figure S3 CCK-8 analysis of trophoblast proliferation capacity after HTR8**/SVneo** cells were transfected with the mimics of all the five miRNAs predicted by online tools
